# The Study of NADPH-Dependent Flavoenzyme-Catalyzed Reduction of Benzo[1,2-c]1,2,5-oxadiazole *N*-Oxides (Benzofuroxans)

**DOI:** 10.3390/ijms151223307

**Published:** 2014-12-15

**Authors:** Jonas Šarlauskas, Lina Misevičienė, Audronė Marozienė, Laimonas Karvelis, Jonita Stankevičiūtė, Kastis Krikštopaitis, Narimantas Čėnas, Aleksey Yantsevich, Audrius Laurynėnas, Žilvinas Anusevičius

**Affiliations:** 1 Institute of Biochemistry of Vilnius University, Mokslininku 12, LT-08662 Vilnius, Lithuania; E-Mails: lina.miseviciene@bchi.vu.lt (L.M.); audrone.maroziene@bchi.vu.lt (A.M.); lkarvelis@f-1.lt (L.K.); jonita.stankeviciute@bchi.vu.lt (J.S.); kastis.krikstopaitis@bchi.vu.lt (K.K.); narimantas.cenas@bchi.vu.lt (N.Č.); audrius.laurynenas@gmail.com (A.L.); 2 Institute of Bioorganic Chemistry, National Academy of Sciences of Belarus, Kuprevich 5/2, 220141 Minsk, Belarus; E-Mail: yantsevich@iboch.bas-net.by

**Keywords:** benzofuroxan, flavoenzyme, redox-cycling, bioreductive conversion, quantitative structure activity relationship, quantum mechanical calculation, DFT

## Abstract

The enzymatic reactivity of a series of benzo[1,2-*c*]1,2,5-oxadiazole *N*-oxides (benzofuroxans; BFXs) towards mammalian single-electron transferring NADPH:cytochrome P-450 reductase (P-450R) and two-electron (hydride) transferring NAD(P)H:quinone oxidoreductase (NQO1) was examined in this work. Since the =N^+^ (→O)O^−^ moiety of furoxan fragments of BFXs bears some similarity to the aromatic nitro-group, the reactivity of BFXs was compared to that of nitro-aromatic compounds (NACs) whose reduction mechanisms by these and other related flavoenzymes have been extensively investigated. The reduction of BFXs by both P-450R and NQO1 was accompanied by O_2_ uptake, which was much lower than the NADPH oxidation rate; except for annelated BFXs, whose reduction was followed by the production of peroxide. In order to analyze the possible quantitative structure-activity relationships (QSARs) of the enzymatic reactivity of the compounds, their electron-accepting potency and other reactivity indices were assessed by quantum mechanical methods. In P-450R-catalyzed reactions, both BFXs and NACs showed the same reactivity dependence on their electron-accepting potency which might be consistent with an “outer sphere” electron transfer mechanism. In NQO1-catalyzed two-electron (hydride) transferring reactions, BFXs acted as more efficient substrates than NACs, and the reduction efficacy of BFXs by NQO1 was in general higher than by single-electron transferring P-450R. In NQO1-catalyzed reactions, QSARs obtained showed that the reduction efficacy of BFXs, as well as that of NACs, was determined by their electron-accepting potency and could be influenced by their binding mode in the active center of NQO1 and by their global softness as their electronic characteristic. The reductive conversion of benzofuroxan by both flavoenzymes yielded the same reduction product of benzofuroxan, 2,3-diaminophenazine, with the formation of *o*-benzoquinone dioxime as a putative primary reductive intermediate, which undergoes a further reduction process. Overall, the data obtained show that by contrast to NACs, the flavoenzyme-catalyzed reduction of BFXs is unlikely to initiate their redox-cycling, which may argue for a minor role of the redox-cycling-type action in the cytotoxicity of BFXs.

## 1. Introduction

The derivatives of benzo[1,2-*c*]1,2,5-oxadiazole *N*-oxide (benzofuroxans, BFXs) comprise an important class of pharmacologically active heterocyclic compounds, which possess antileukemic, immunosuppressive, anti-infective, antibacterial, antifungal, insecticidal, and antiparasitic (antiplasmodial and trypanocidal) activities ([1,2,3,4,5,6], and references therein). In addition, some of their representatives may also be used as explosive materials ([7], and references therein). However, despite the multiple activities of BFXs, their molecular action mechanisms are still not well understood, except for the characterization of several of their representatives as the inhibitors of monoamine oxidase [8] as vasodilating substances ([1,9] and references therein), and potential alkylating agents for cellular thiols [10,11].

**Figure 1:**
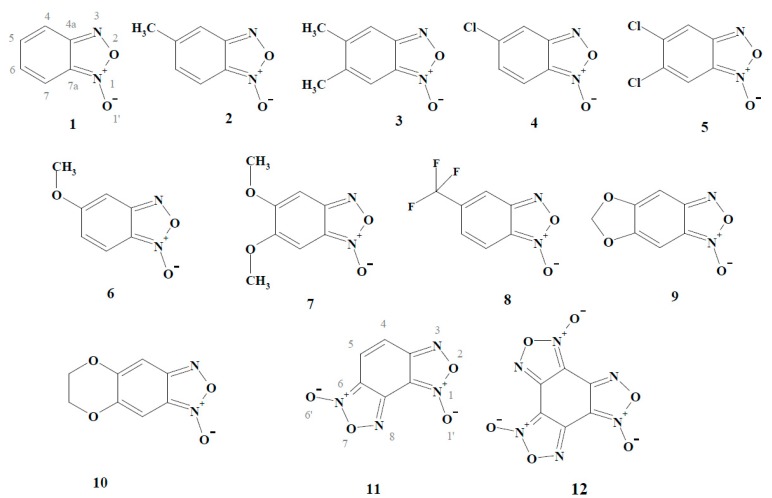
Structural formulas of benzofuroxan compounds used in the study.

On the other hand, the redox activity of BFXs may also be regarded as an important factor in their (cyto)toxicity, because the =N^+^(→O)O^−^ moiety of BFXs (Figure 1) bears some similarity to a redox active nitro-group ([12,13] and references therein). Typically, the cytotoxic activity of nitroaromatic compounds (NACs) is associated with their single-electron enzymatic reduction, leading to the formation of their anion-radicals ([14,15,16,17,18], and references therein). The latter are then rapidly re-oxidized by oxygen with the formation of superoxide and, subsequently, other reactive oxygen species (ROS), causing oxidative stress-type cytotoxicity ([14,15,16,17,18] and references therein). In contrast to the single-electron enzymatic reduction of NACs, their two(four)-electron enzymatic reduction results in the formation of hydroxylamines which alkylate DNA ([19] and references therein). In this context, there exist some data on the (bio)reductive transformation of BFXs, which may be important in the manifestation of their toxicity: (i) the formation of their free radicals, detected in hybrid compounds bearing BFXs as pharmacophors in the *T. cruzi* microsomal fraction, suggesting that BFXs might be able to produce oxidative stress in parasites [4]; (ii) the reduction of some BFXs to corresponding nitroaniline derivatives by oxyhemoglobin [20]; and (iii) the reduction of benzofuroxan to *o*-benzoquinone dioxime and 2,3-diaminophenazine in cytosolic and microsomal fractions of rat liver [21]. However, these data remain poorly interrelated and do not provide a quantitative insight into the mode of action of BFXs.

In this study, we have examined the reactivity of a series of BFXs (Figure 1) towards single-electron transferring flavoenzyme NADPH:cytochrome P-450 reductase (P-450R; EC 1.6.2.4) and two-electron (hydride) transferring flavoenzyme NAD(P)H:quinone oxidoreductase (DT-diaphorase, NQO1; EC 1.6.99.2) which are known to be the main participants in the cellular redox-cycling and/or bioreductive activation of numerous groups of redox active cytotoxic agents ([14,15,16,17,18] and references cited therein). Owing to the =N^+^(→O)O^−^ moiety of BFXs, one may expect that BFXs act as electron-accepting oxidants in their flavoenzyme-catalyzed reduction reactions such as NACs, whose reduction mechanisms by these and other related flavoenzymes have been extensively researched. As a rule, the reactivity of NACs towards P-450R or other single-electron transferring flavoenzymes increases with an increase in their electron-accepting potency expressed in terms of their single-electron reduction potential (E^1^_7_) or quantum mechanically calculated electron-accepting parameter values ([14,15,16,17,18] and references therein). These reactions follow an “outer-sphere” electron transfer (ET) mechanism [22], *i.e.*, ET proceeds with a minimal electronic coupling between the reactants with their structural features having a negligible impact on the process. In contrast to the single-electron transferring reactions, the mechanisms of two-electron (hydride) reduction of NACs by NQO1 and other related two-electron transferring flavoenzymes, such as oxygen insensitive nitroreductases, are still not thoroughly understood. However, some defined dependences of the reactivity of NACs towards the latter enzymes on E^1^_7_ of the compounds have been associated with the possible involvement of a multistep single-electron transfer in their two-electron reduction reactions ([23], and references therein).

In this work, we have found that BFXs acted as relatively efficient substrates for single-electron transferring P-450R as well as two-electron transferring NQO1. The reduction of the compounds by both flavoenzymes was concomitantly accompanied by the formation of their reductive intermediate(s). During their reduction, the rate of O_2_ uptake was much lower than that of NADPH oxidation except for annelated BFXs (Compounds 11–12, Figure 1), whose reduction by both enzymes was accompanied by the concomitant production of peroxide. During the study of the possible existence of quantitative structure-activity relationships (QSARs) for the reactivity of BFXs together with NACs, their electron-accepting potency was evaluated by quantum mechanical computation, and, additionally, their global reactivity indices, such as global electrophilicity (ϖ) and global softness (S), were assessed by applying the conceptual density functional theory (DFT) approach. It was found that in single-electron transferring P-450R-catalyzed reactions, BFXs and NACs demonstrated the same dependence upon their electron-accepting potency, whereas in two-electron transferring NQO1-catalyzed reactions, BFXs acted as more efficient substrates than NACs. The data obtained suggested that compared to NACs, the higher reactivity of BFXs towards NQO1 could be influenced by several factors such as their specific binding site in the enzyme active center, which may differ from that of NACs, and/or the electronic character of the compounds expressed in terms of their global softness. The data show that P-450R and NQO1 can be responsible for the bio-reductive transformation of BFXs or related structure compounds bearing BFXs.

## 2. Results and Discussion

### 2.1. The Computational Study of the Electron-Accepting Properties of BFXs

At the initial stage of this study, we characterized the electron accepting properties of BFXs by means of a computational study and compared them with those of (poly)nitrobenzene compounds as model nitroaromatic compounds (NACs), whose energetic of single-electron reduction, *i.e.*, the single-electron reduction potentials (E^1^_7_, the potential of NACs/NACs^−.^ redox couple) are well defined (Table 1) ([16,24], and references therein). In this study, we carried out gas-phase computations using B3LYP functional method in conjunction with 6-311+G(d,p) basis set (more details are given in the Experimental Section).

Table 1 lists the calculated energies of the vertical electron affinity (VEA) of the compounds, denoting the difference in Gibbs free energy between their neutral and anionic radical species at a fixed geometry of their neutral state, and the adiabatic electron affinity (AEA), reflecting the difference in Gibbs free energy between their neutral and anion radical species when both are in their optimized states. The experimentally defined E^1^_7_ of NACs satisfactorily correlated with their VEA (R^2^ = 0.938, F_1,3_ = 45.719, *p* < 0.005) and to a slightly greater extent with AEA (R^2^ = 0.946, F_1,3_ = 53.110, *p* < 0.005), suggesting that E^1^_7_ might be associated with the formation of their anion radical species at their relaxed states. The calculation of BFXs (Compounds 1–10) showed the expected trend due to their substituents, *i.e.*, electron-withdrawing groups made EAs higher, while electron-donating ones had the opposite effect. For annelated (explosive) BFXs (Compounds 11–12), EAs increased with an increase in the number of furoxan rings. The fair correlation of AEA *versus* VEA of BFXs together with NACs was defined: AEA = (0.269 ± 0.075) + (0.963 ± 0.047)VEA (R^2^ = 0.965, F_1,15_ = 414.607, *p* < 0.0001), where the positive intercept value of ≈0.27 eV may reflect the approximate difference between their AEA and VEA parameter values, implying that upon accepting an electron, like NACs, BFXs undergo significant relaxation to their equilibrated state.

**Table 1:** The experimental single-electron reduction potentials (E^1^_7_) of nitro-aromatic compounds (NACs) and the quantum mechanical indices of benzofuroxans (BFXs) and NACs as calculated by using the B3LYP/6-311+G(d,p) method: the vertical electron affinity (VEA), the adiabatic electron affinity (VEA), the global softness (*S*), the global electronegativity (χ), and the global electrophilicity (ϖ). The E^1^_7_ values are taken from [16,24].

No.	Compound	E^1^_7_ (V)	VEA (eV)	AEA (eV)	*S* (eV^−1^)	Χ (eV)	ϖ (eV)
	*Benzofuroxans*						
1.	Benzofuroxan		1.241	1.433	0.142	4.799	3.262
2.	5-Methylbenzofuroxan		1.146	1.348	0.140	4.668	3.040
3.	5,6-Dimethylbenzofuroxan		1.093	1.202	0.141	4.527	2.885
4.	5-Chlorobenzofuroxan		1.524	1.729	0.142	5.029	3.580
5.	5,6-Dichlorobenzofuroxan		1.760	1.962	0.146	5.150	3.877
6.	5-Methoxybenzofuroxan		1.051	1.296	0.136	4.595	2.897
7.	5,6-Dimethoxybenzofuroxan		0.815	1.082	0.137	4.274	2.499
8.	5-Trifluorobenzofuroxan		1.734	1.962	0.143	5.253	3.928
9.	5,6-Methylenedioxybenzofuroxan		0.990	1.240	0.137	4.514	2.795
10.	5,6-Ethylenedioxybenzofuroxan		1.035	1.282	0.146	4.419	2.852
11.	Benzodifuroxan		1.710	1.992	0.125	5.382	3.621
12.	Benzotrifuroxan		2.384	2.507	0.121	5.947	4.278
	*Nitrobenzenes*						
13.	Nitrobenzene	−0.485	0.991	1.304	0.099	5.435	2.933
14.	1,2-Dinitrobenzene	−0.287	1.817	2.093	0.097	6.192	3.548
15.	1,3-Dinitrobenzene	−0.345	1.703	2.040	0.102	5.902	3.694
16.	1,4-Dinitrobenzene	−0.257	2.212	2.510	0.105	6.332	4.196
17.	2,4,6-Trinitrotoluene	−0.253	2.370	2.555	0.102	6.378	4.130

In addition, Table 1 lists a set of global reactivity indices of the compounds, *i.e.*, the global softness index (*S*), the absolute electronegativity index (χ), and the global electrophilicity index (ϖ). These indices were assessed within the framework of conceptual density functional theory (DFT) applying Koopmans’ theorem in terms of the energies of the highest occupied molecular orbital (E_HOMO_) and the lowest unoccupied molecular orbital (E_LUMO_) ([25,26,27,28,29], and references therein) (Equations (1)–(3)):

*S* = 1/2η = 1/{2( E_LUMO_ − E_HOMO_)} (1)

χ = −(E_LUMO_ + E_HOMO_)/2 (2)

ϖ = χ^2^*S* = (E_LUMO_ + E_HOMO_)^2^/{8(E_LUMO_ − E_HOMO_)} (3)

In accordance with Janak’s approximation [25], there is a connection between the vertical ionization potential (VIP) and E_HOMO_ (VIP ≈ −E_HOMO_) as well as between the vertical electron affinity and E_LUMO_ (VEA ≈ −E_LUMO_). Thus the trend of DFT global indices of the compounds, obtained in terms of LUMO and HOMO eigenvalues, is expected to be almost the same as that when using their VIP and VEA values. The global *S* index, which bears an inverse relationship with the global hardness index (*S* = 1/2η), is a function of LUMO/HOMO energy gap (Equation (1)). It may serve as a rough criterion for the thermodynamic stability of the compounds and can be used for their reactivity prediction,* i.e.*, the softer molecule, which has a smaller LUMO/HOMO gap, may undergo an easier rearrangement in charge density, and hence it could be predicted to be more reactive than the harder ones ([27,29], and references therein). In addition, the global softness (or hardness) index might be used in probing the aromatic character of organic compounds, *i.e.*, compounds which have a smaller LUMO/HOMO gap might be considered to have less aromatic character compared to those with a larger LUMO/HOMO gap ([30] and references therein). The global ϖ index (Equation (3)), as a function of global electronegativity (χ) (Equation (2)) and global softness (Equation (1)), is frequently treated as a propensity of the compounds to accept the relative number of electrons ([26], and references therein). It is generally anticipated that the global ϖ index is to some extent related to EAs, but cannot be equal to them [28]. This index has been successfully applied for prediction of the electron-accepting potency for single- and/or two-electron (hydride) reduction of redox active xenobiotics such as NACs and quinones [31,32,33,34]. Note that for neutral systems, this index has been found to be almost insensitive to solvent effects ([31] and references therein).

As shown in Table 1, the assessed global *S* index values of the whole set of BFXs (0.146–0.121 eV^−1^) were markedly higher compared to those of *NACs* (0.097–0.105 eV^−1^), suggesting that upon their reduction, BFXs with a smaller LUMO/HOMO gap may undergo an easier rearrangement in charge density and thus an easier conversion to their reductive intermediate(s). BFXs (Compounds 1–10) spanned within a relatively small variation in their *S* values (0.146–0.137 eV^−1^), insignificantly depending upon their electron-withdrawing or -donating groups, while markedly lower softness was assessed for annelated BFXs, 0.125 eV^−1^ (compound 11) and 0.121 eV^−1^ (compound 12). The *S* index values of NACs almost did not depend upon the number of nitro-groups and their positions. It may be noted that almost the same tendency was previously obtained for a series of (poly)nitroaromatic compounds, as computed by means of the DFT approach [33]. The global ϖ index values of BFXs and NACs were determined to correlate well with their VEA (R^2^ = 0.941, F_1,15_ = 239.076, *p* < 0.0001) and to a lesser extent with their AEA (R^2^ = 0.885, F_1,15_ =115.800, *p* < 0.0001). The data obtained show that the assessed electron accepting potency of BFXs expressed in terms of their EAs and global ϖ index values varied almost in the same range as that of NACs considered in this work.

In addition, we predicted the local electrophilic sites of BFXs by performing the calculation of their electrophilic Fukui index (F^+^_k_) values, which may reflect the tendency of k-atom to accept the nucleophile (an electron or a hydride ion) at the initial stage of BFXs' reduction. The F^+^_k_ values were assessed by the frontier molecular orbital (FMO) approach as described concisely in the Experimental Section. The calculation showed that in general the highest F^+^_k_ values of BFXs reside upon N-1 atom of the =N^+^ (→O)O- moiety, providing the electrophilic character for the furoxan fragment in an approximate order: F^+^_N-3_ ≥ F^+^_O-1'_ > F^+^_O-2_. In addition, for BFXs (Compounds 1–10), the relatively high F^+^_k_ values were distributed on C-4 and C-7 atoms of the benzene ring. One may note the exceptions for Compound 5, whose highest F^+^_k_ values reside upon the C-4 and C-7 atoms of the benzene ring, as well as for the annelated BFX Compound 11, whose largest F^+^_k_ values reside upon the C-4 and C-5 atoms of the annelated benzene ring.

### 2.2. The Study of Enzymatic Reactivity of BFXs

#### 2.2.1. P-450R-Catalyzed Reduction of BFXs

**Figure 2:**
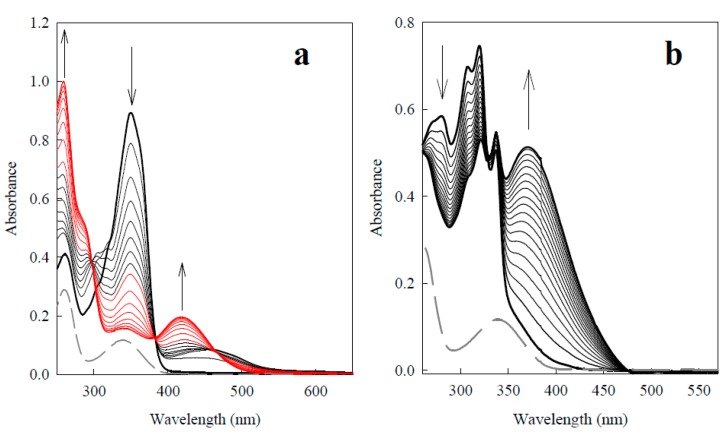
UV-VIS absorbance spectra of reduction of 100 μM benzofuroxan (Compound 1) (**a**) and reduction of 100 μM benzodifuroxan (Compound 11) (**b**) in the presence of NADPH-regeneration system (gray dashed curves) by 250 and 50 nM of P-450R, respectively. The spectra of benzofuroxan were recorded every 10 min at the first stage of the reaction (black curves), and subsequently scanned in 20-min intervals (red curves). The reduction of benzodifuroxan was scanned in 4-min intervals. The arrows indicate the directions of absorbance changes.

Upon studying the reduction kinetics of BFXs by single-electron transferring P-450R, the reactions were initially examined in the presence of the NADPH-regeneration system (10 U/mL glucose-6-phosphate dehydrogenase, 10 mM glucose-6-phosphate, and 15–20 μM NADPH). As shown in Figure 2, the reductions of benzofuroxan and benzodifuroxan were accompanied by the UV-VIS absorbance changes, thus indicating that the reduction of the compounds results in the concomitant formation of their reductive product(s). The absorbance changes in NADPH-regeneration system were also obtained for the reduction of the whole set of BFXs used in this study (data not shown).

During the study of the redox-cycling ability of BFXs, it was determined that the reduction of BFXs (Compounds 1–10) by P-450R was accompanied by slow O_2_ uptake, at the rate close to that of the intrinsic NADPH-oxidase activity of the enzyme (0.11 ± 0.02 s^−1^), whereas the enzymatic reduction of annelated BFXs (compounds 11–12) was followed by rapid O_2_ uptake almost at the same rate as that of the NADPH oxidation. The latter reactions were suppressed by 40%–45% by catalase (30 µg/mL), while the addition of superoxide dismutase (SOD, 100 U/mL) to the reaction mixture had no apparent effect on the reactions. These data imply that BFXs (compounds 1–10) are reduced to the relatively stable (oxygen-insensitive) intermediate(s), whereas annelated BFXs are converted to less stable intermediate(s), being readily oxidized by O_2_ producing peroxide. This is in sharp contrast to NACs, whose reduction by P-450R or other related single-electron transferring flavoenzymes to nitro radicals is accompanied by their concomitant reoxidation by O_2_ producing superoxide, thus giving rise to futile redox-cycling of the compounds ([14,15,16,17,18], and references therein).

The determined initial rates of the reduction of BFXs by P-450R showed incomplete hyperbolic or linear dependences on concentrations of BFXs, varying in the range of 12.5–400 µM (data not shown). Due to the limited solubility and high optical density of the compounds, their higher concentrations were not used to attain enzyme saturation for accurate determination of k_cat_ values. Therefore, only the values of the apparent second rate constant (k_cat_/K_m_) were defined by applying re-parameterized Michaelis-Menten or linear rate expressions (Equations (7) and (8), see the Experimental Section). As shown in Table 2, the determined k_cat_/K_m_ values of BFXs together with those of NACs varied by several orders of magnitude.

**Table 2:** The apparent second-order rate (k_cat_/K_m_) constants of reduction of BFXs and NACs by NADPH:cytochrome P-450 reductase (P-450R), pH 7.0, 25°C. All kinetic measurements were performed at fixed concentration of NADPH (150 µM).

No.	Compound	k_cat_/K_m_ (M^−1^·s^−1^)
	*Benzofuroxans*	
1.	Benzofuroxan	1.4 ± 0.2 × 10^3^
2.	5-Methylbenzofuroxan	2.1 ± 0.1 × 10^3^
3.	5-Dimethylbenzofuroxan	7.1 ± 0.8 × 10^2^
4.	5-Chlorobenzofuroxan	3.2 ± 0.2 × 10^3^
5.	5,6-Dichlorobenzofuroxan	4.3 ± 0.3 × 10^4^
6.	5-Methoxybenzofuroxan	7.3 ± 0.3 × 10^2^
7.	5,6-Dimethoxybenzofuroxan	5.6 ± 0.5 × 10^2^
8.	5-Trifluorobenzofuroxan	1.1 ± 0.1 × 10^4^
9.	5,6-Methylendioxybenzofuroxan	1.1 ± 0.1 × 10^3^
10.	5,6-Ethylendioxybenzofuroxan	4.5 ± 0.5 × 10^3^
11.	Benzodifuroxan	1.0 ± 0.1 × 10^4^
12.	Benzotrifuroxan	2.9 ± 0.2 × 10^5^
	*Nitrobenzenes*	
13.	Nitrobenzene	2.8 ± 0.3 × 10^3^
14.	1,2-Dinitrobenzene	2.0 ± 0.2 × 10^5^
15.	1,3-Dinitrobenzene	4.9 ± 0.4 × 10^4^
16.	1,4-Dinitrobenzene	2.6 ± 0.3 × 10^6^
17.	2,4,6-Trinitrotoluene	2.3 ± 0.2 × 10^7^

**Figure 3:**
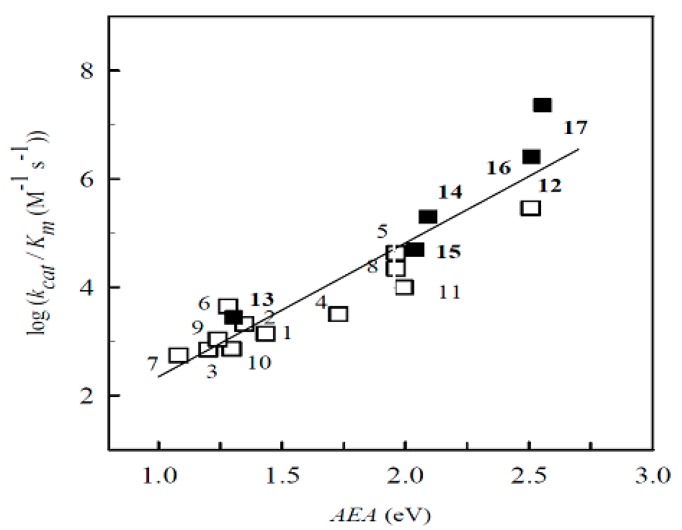
The dependence of reactivity expressed as log of the apparent second-order rate constants (k_cat_/K_m_) of reduction of BFXs (Compounds 1–12, open squares) and NACs (Compounds 13–17, black squares) by NADPH:cytochrome P-450 reductase (P-450R) upon their adiabatic electron affinity (AEA), calculated by using B3LYP/6-311+G(d,p) method.

The log k_cat_/K_m_ exhibited a well expressed dependence on their electron-accepting potency: R^2^ = 0.884, F_1,11_ = 74.712, *p* < 0.0001 (VEA) or R^2^ = 0.887, F_1,11_ = 78.41, *p* < 0.0001 (AEA) (Figure 3), and correlated well with their global ϖ index values (R^2^ = 0.803, F_1,11_ = 40.631, *p* < 0.0001). Importantly, the reactivity of BFXs correlated well with that of NACs with their VEA (R^2^ = 0.833, F_1,15_ = 75.998, *p* < 0.0001) or AEA (R^2^ = 0.844, F_1_,_15_ = 81.399, *p* < 0.0001), and to a markedly lesser extent with their global ϖ values (R^2^ = 0.698, F_1,15_ = 34.789, *p* < 0.0001). This might be consistent with an “outer-sphere” electron transfer (ET) mechanism [23], where the reaction rate is determined primarily by the difference of redox potentials of the reagents as an ET driving force. This reaction may take place at the initial stage of the reductive conversion of BFXs, with the first electron transfer as a rate-limiting stage. Note that the reactivity of BFXs was also found to be dependent upon their quantum mechanically assessed electron-accepting parameter values towards another single-electron transferring flavoenzyme rat neuronal nitric oxide synthase (nNOX, EC 1.14. 13.39) (unpublished data).

#### 2.2.2. NQO1-Catalyzed Reduction of BFXs

**Figure 4:**
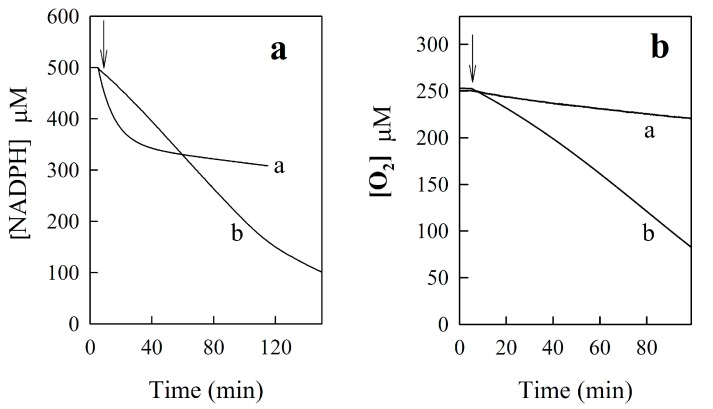
Time-course oxidation of excess amount NADPH (500 µM) (**a**) and oxygen uptake (**b**) during the NQO1-catalyzed reduction of 50 μM 5-chlorobenzofuroxan (curve a) and 50 µM benzodifuroxan (curve b). Arrows indicate the introduction of 100 nM NQO1.

During the reduction of BFXs by two-electron transferring NQO1 in NADPH-regeneration system, the character of their UV-VIS absorbance changes was almost the same as in P-450R-catalyzed reactions, suggesting that the same reductive product(s) should be formed (data not shown). The reactions were inhibited by 1 μM dicumarol, a specific inhibitor of NQO1 [20,35,36]. Like in P-450R-catalyzed reactions, the reduction of BFXs (Compounds 1–10) by NQO1 was followed by the slow consumption of O_2_ at the rate close to that of the intrinsic NADPH-oxidase activity of NQO1 (0.05 ± 0.02 s^−1^), while the reduction of annelated BFXs (Compounds 11–12) was accompanied by O_2_ uptake, whose rate was almost the same as that of NADPH oxidation. In parallel experiments, the BFXs-mediated reduction of cytochrome *c* (50 μM) was examined, applying cytochrome *c* as the terminal single-electron acceptor. Note that this assay was not used for the reduction of BFXs by P-450R since this enzyme catalyzes the direct reduction of cytochrome *c*, with k_cat_/K_m_ of 1.6 × 10^6^ M^−1^·s^−1^. In the NQO1-catalyzed reduction of BFXs (Compounds 1–10), the rate of the reduction of cytochrome *c* accounted for 35%–40% of NADPH oxidation rate. The introduction of SOD to the reaction mixture suppressed the reaction by 15%–20%, which may indicate the partial formation of superoxide during the reaction. Meanwhile, the reduction of annelated BFXs (Compounds 11–12) was accompanied by the reduction of cytochrome *c* accounting for 190%–200% of NADPH oxidation rate. The reaction was suppressed by 35%–40% by introduction of SOD to the reaction mixture, confirming that annelated BFXs are reduced to less stable reductive intermediate(s). The reduction of cytochrome *c* is unlikely to be related to the formation of anion radicals of BFXs, but may be caused by the direct single-electron oxidation of reductive intermediate(s) by cytochrome *c* to their radical species, which can be partially oxidized by O_2_ producing peroxide. In addition, we checked the redox-cycling ability of certain BFXs by recording the progress curves of oxidation of the excess amount of NADPH and, in parallel, oxygen uptake. As shown in Figure 4a (curve a), the NQO1-catalyzed oxidation of 500 µM NADPH by 50 µM 5-chlorobenzofuroxan, monitored at 305 nm (∆ε_305_ = 0.49 mM^−1^·cm^−1^), followed the biphasic kinetics: approximately three equivalents of NADPH were oxidized in the first (fast) phase, and the rate of NADPH oxidation in the second (slow) phase was almost the same as that of the intrinsic NADPH-oxidase activity of NQO1. This suggests that the 6-electron reduced product of the compound may be formed. The reduction of the compound was followed by slow O_2_ uptake (Figure 4b, curve a) at a rate close to that of the oxidase activity of the enzyme. Less obvious biphasic kinetic was observed for the reduction of the compound by P450R (data not shown) which is probably due to the lower reactivity of P-450R and higher intrinsic NADPH-oxidase activity of the enzyme, as compared to those of NQO1. Meanwhile, the NQO1-catalyzed oxidation of excess NADPH by 50 µM annelated BFX, benzodifuroxan (compound 11), measured at isosbestic point of its absorbance spectra at 340 nm (∆ε_340_ = 6.22 mM^−1^·cm^−1^), followed almost a single continuous phase (Figure 4a, curve b), and the exhaustion of excess amount of O_2_ was observed (Figure 4b, curve b). The same was obtained for the P-450R-catalyzed reduction of benzodifuroxan (data not shown).

The determined initial rates of the reduction of the whole set of BFXs by NQO1 followed the complete hyperbolic dependence upon concentrations of the compounds, varying in the range of 12.5–400 µM (data not shown). The determined catalytic constant (k_cat_) and k_cat_/K_m_ ratio values of BFXs together with those of NACs are listed in Table 2.

The kinetic parameters of the reduction of BFXs did not depend upon the varying concentrations of NADPH, 10–500 µM, indicating that the apparent K_m_ value of NADPH (K_m(NADPH)_) is rather low. The same was previously observed for the NQO1-catalyzed reduction of low reactive quinones and NACs. It should be noted that a low K_m(NADPH)_ value does not imply that NADPH binds to the oxidized enzyme form with high affinity. The steady-state and transient kinetic studies showed that NQO1 follows a ping-ping reaction scheme, and that the rate limiting stage is the reduction of substrates by the reduced enzyme form [35,36,37]. The transient kinetic studies of reduction of NQO1 by NADPH revealed no evidence for the complex formation between the oxidized enzyme form and NADPH. It was assumed that if such a complex does exist, it must be of low affinity, with K_d_ value >0.5 mM [36]. The previously performed steady-state kinetics for the reduction of quinones (highly reactive oxidants) and NACs (relatively low reactive oxidants) showed that K_m(NADPH)_ increases with an increase in reactivity of the compounds. This enables us to suggest that in the catalytic cycle of NQO1, the enzyme follows apparent second order rate kinetics with respect to NADPH oxidation and thus K_m(NADPH)_ becomes the ratio of the catalytic constant (k_cat_) of reduction of the compounds and the apparent second-order rate constant (k_cat_/K_m(NADPH)_) of the oxidation of NADPH: K_m(NADPH)_ = k_cat_/(k_cat_/K_m(NADPH)_). As determined by steady-state kinetics in this and our previous works for the quinone reductase reactions of NQO1 [36], k_cat_/K_m(NADPH)_ varies in the range of 1.5–2.0 × 10^7^ M^−1^·s^−1^. Thus even for more reactive BFXs (Table 3), apparent K_m(NADPH)_ can be predicted to be lower than 1 µM.

**Table 3:** The catalytic (k_cat_) and the apparent second-order rate (k_cat_/K_m_) constants of reduction of BFXs and NACs by NAD(P)H:quinone oxidoreductase (NQO1), pH 7.0, 25 °C. All kinetic measurements were performed at fixed concentration of NADPH (150 µM).

No.	Compound	k_cat_ (s^−1^)	k_cat_/K_m_ (M^−1^·s^−1^)
	*Benzofuroxans*		
1.	Benzofuroxan	11.0 ± 1.5	8.4 ± 0.3 × 10^4^
2.	5-Methylbenzofuroxan	2.4 ± 0.4	1.4 ± 0.1 × 10^4^
3.	5-Dimethylbenzofuroxan	1.8 ± 0.2	4.7 ± 1.1 × 10^3^
4.	5-Chlorobenzofuroxan	5.6 ± 0.6	6.2 ± 0.5 × 10^4^
5.	5,6-Dichlorobenzofuroxan	6.9 ± 0.1	1.3 ± 0.1 × 10^5^
6.	5-Methoxybenzofuroxan	4.4 ± 0.1	2.5 ± 0.4 × 10^4^
7.	5,6-Dimethoxybenzofuroxan	1.1 ± 0.1	1.4 ± 0.3 × 10^4^
8.	5-Trifluorobenzofuroxan	7.6 ± 0.9	2.5 ± 0.4 × 10^5^
9.	5,6-Methylendioxybenzofuroxan	1.2 ± 0.4	4.6 ± 0.9 × 10^3^
10.	5,6-Ethylendioxybenzofuroxan	0.7 ± 0.1	8.1 ± 1.7 × 10^3^
11.	Benzodifuroxan	2.3 ± 0.4	1.6 ± 0.3 × 10^4^
12.	Benzotrifuroxan	2.6 ± 0.3	3.8 ± 0.4 × 10^4^
	*Nitrobenzenes*		
13.	Nitrobenzene	<0.1	2.4 ± 0.2 × 10^1^
14.	1,2-Dinitrobenzene	<0.1	6.6 ± 0.7 × 10^2^
15.	1,3-Dinitrobenzene	0.3	7.4 ± 0.7 × 10^2^
16.	1,4-Dinitrobenzene	0.6 ± 0.1	2.4 ± 0.1 × 10^3^
17.	2,4,6-Trinitrotoluene	1.0	6.7 ± 0.7 × 10^2^

**Figure 5:**
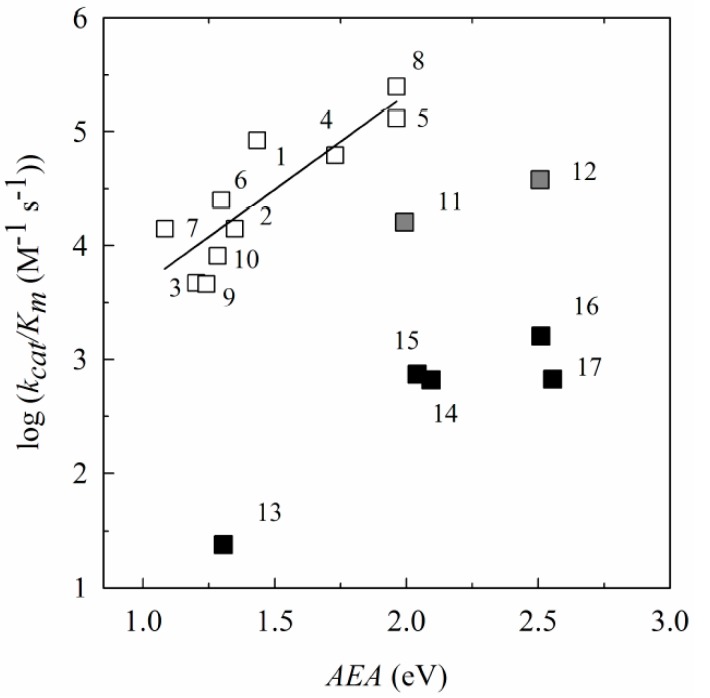
The dependence of the log of the apparent second-order rate constants (k_cat_/K_m_) of reduction of BFXs (compounds 1–10, open squares), annelated BFXs (Compounds 11–12, filled gray squares) and model NACs (Compounds 13–17, filled black squares) by NAD(P)H:quinone oxidoreductase (NQO1) upon their AEA values, assessed using B3LYP/6-311+G(d,p) method. The linear correlation is defined for BFXs, omitting annelated BFX Compounds 11 and 12.

As shown in Table 2 and Table 3, NQO1 catalyzed the reduction of BFXs more efficiently than P-450R, as reflected in terms of their k_cat_/K_m_ values, except for annelated BFXs (Compounds 11–12), whose reduction by P-450R proceeded with higher rates, as compared to the reduction of the compounds by NQO1. As can be seen from the plotted log k_cat_/K_m_ of the compounds *versus* their AEA (Figure 5), the reactivity of BFXs was almost by two orders of magnitude higher than that of NACs, implying that in contrast to P-450R, NQO1 possesses a markedly higher preference for BFXs. The log k_cat_/K_m_ of BFXs increased with an increase in their electron-accepting parameter values, omitting annelated BFXs, whose reactivity was markedly lower, compared with their electron-accepting potency.

The log k_cat_/K_m_ of higher reactive BFXs (Compounds 1–10) moderately correlated with their VEA (R^2^ = 0.702, F_1,8_ = 19.105, *p* < 0.0024) and to a greater extent with their AEA (R^2^ = 0.750, F_1,8_ = 23.972, *p* < 0.0013) (Figure 4) or ϖ index values (R^2^ = 0.751, F_1,8_ = 24.159, *p* < 0.0012), suggesting that the reactivity of BFXs towards two-electron transferring NQO1 is determined by their electron-accepting potency. However, the data show that the reduction of the compounds by NQO1 can also be influenced by other factors. Note that the rough influence of the structural features of a long series of structurally diverse quinones and NACs, expressed in terms of their Van der Waals volume (VdWvol), on their reactivity towards NQO1 has been previously defined ([19,20,37], and references therein). The reactivity of BFXs and NACs used in this study was found to be independent of their VdWvol used as a sole or additional descriptor to EAs or ϖ (data not shown), as it appeared to be an irrelevant factor in their reactivity. The same results were obtained when applying the calculated hydrophobicity parameter (clog P) values (data not shown). We then attempted to do an analysis with a two-parametric regression by using the global DFT quantities of the compounds, *i.e.*, their global softness index was introduced as an additional variable to their electrophilicity index. Moderate regression was defined for the whole set of BFXs (R^2^ = 0.682, R^2^_adj_ = 0.611, F_2,9_ = 9.652, *p* < 0.006):

log k_cat_/K_m_ = −(3.036 ± 2.328) + (0.873 ± 0.203) ϖ + (33.158 ± 14.584) *S* (4)

whereas a markedly higher regression was obtained for the whole set of BFXs together with NACs (R^2^ = 0.877, R^2^_adj_ =0.860, F_2,14_ = 29.010, *p* < 0.0001):

log (k_cat_/K_m_) = −(5.682 ± 1.378) + (0.856 ± 0.228)ϖ + (51.81 ± 6.852)*S* (5)

indicating that the enzymatic reactivity of the compounds tends to increase with an increase in their global softness as an intrinsic reactivity descriptor.

**Figure 6:**
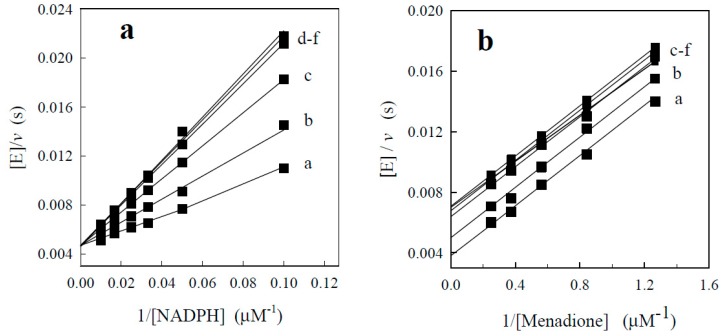
The inhibition of NQO1 by benzofuroxan with respect to the quinone reductase reaction of NQO1 (**a**) The dependence of the enzymatic rate on concentrations of NADPH at fixed concentration of menadione (4 µM) and fixed concentrations of benzofuroxan: 0 µM (a), 25 µM (b), 50 µM (c), 100 µM (d), 200 µM (e), and 300 µM (f); (**b**) The dependence of the enzymatic rate on varied concentrations of menadione at fixed concentration of NADPH (40 µM).

In an attempt to predict other possible factors which may influence the reactivity of BFXs, we examined the inhibition of NQO1 by BFXs in NAD(P)H:quinone reductase reaction of NQO1 by measuring the initial rates of menadione-mediated reduction of cytochome *c.* This may provide some information about the character of the compounds binding to the active center of the enzyme ([19,20], and references therein). BFXs showed the same type of inhibition as previously defined for NACs ([19,20], and references therein): competitive (specific) inhibition with respect to NADPH (increasing the slopes (K_m(NADPH)_/k_cat_) without affecting the intercept ([E]/V_max_) of the double reciprocal plots of [E]/*v versus* 1/NADPH (Figure 6a), and uncompetitive inhibition with respect to quinone (affecting the intercept of [E]/v *versus* 1/quinone, without altering the slopes (K_m(quinone)_/k_cat_) (Figure 6b). This clearly indicates that in the quinone reductase reaction of NQO1 BFXs compete with NADPH for the active site of the oxidized enzyme form by k_cat_/K_m(NADPH)_ or increasing apparent K_m(NADPH)_.

**Figure 7:**
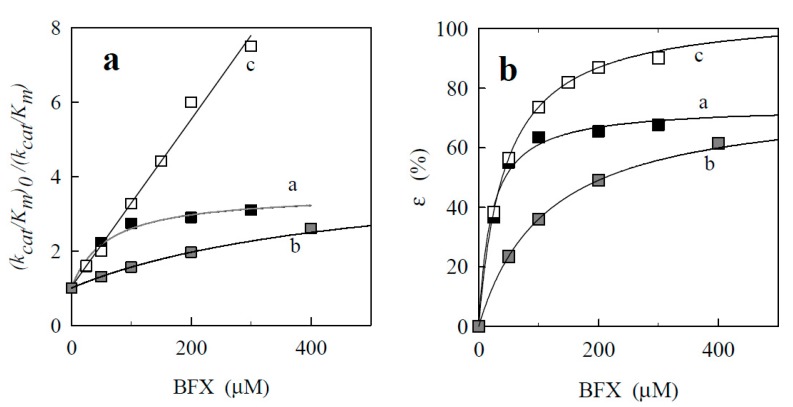
(**a**) The dependence of (k_cat_/K_m_)_0_/(k_cat_/K_m_) ratio on concentrations of BFXs (k_cat_/K_m0_ and k_cat_/K_m_ denote the apparent second rate constants of NADPH oxidation in the absence and presence of BFXs as inhibitors, respectively): benzofuroxan (curve a), 5,6-methylendioxybenzofuroxan (curve b), and benzotrifuroxan (curve c); (**b**) The inhibition degree (ε) of NQO1 on varied concentrations of BFXs.

As illustrated by Dixon’s plots for certain BFXs (Figure 7a), more reactive BFXs behaved as incomplete (non-linear) inhibitors, while annelated BFXs, which are less reactive compounds, acted as complete (linear) inhibitors. Note that in control experiments, the incomplete inhibition was not related to BFXs-dependent reduction of cytochrome *c*. The complete (linear) inhibition type was previously defined for NACs ([20], and references therein) in quinone reductase reaction of NQO1; their inhibition constant (K_i_) values are given in Table 4. As shown in Figure 7b, the assessed inhibition degree (ε) of BFXs showed hyperbolic dependence upon their varied concentrations. Their maximal inhibition degrees (ε_max_) and K_i_ values, defined from the hyperbolic expression of Equation (11) (for details see the Experimental Section), are listed in Table 4. For more reactive BFXs (Compounds 1–10), the estimated ε_max_ values varied in the range of 70%–90% , and the K_i_ values of the whole set of BFXs were generally lower than those of NACs, suggesting that BFXs may bind to the enzyme active center more efficiently than NACs.

The defined incomplete character of inhibition of more reactive BFXs (Compounds 1–10) suggests that they may bind at the binding site, which partially overlaps with that of NADPH and differs from that of NACs and probably of annelated BFXs, which compete with NADPH for the binding site which might be the same or overlapping. In accordance with the previous studies ([19,20,37] and references therein), NACs occupy the adenosine phosphate-binding region of NADPH substrate, which is rather remote from the isoalloxazine ring of FAD. Thus the higher reactivity of BFXs (Compounds 1–10), as compared to NACs or annelated BFXs, might be partially due to their specific yet unidentified binding site in the active center of NQO1. However, uncertain correlations were observed between the enzymatic reactivity of BFXs and their log K_i_ used as a sole parameter or an additional variable to electron-accepting (EAs or ϖ) parameter values (data not shown).

Although the understanding of NQO1-catalyzed reduction mechanism of BFXs is still far from being complete, the defined QSARs (Equations (4) and (5)) suggest that the reactivity of the compounds can be determined by their electron-accepting potency and influenced by other factors such as their binding character in the enzyme active center and the electronic properties of the compounds. One may suppose that the initial stage of the reduction of BFXs might be consistent with the multistep (e^−^, H^+^, e^−^ or e^−^, H^.^) hydride transfer mechanism which may proceed through a charge transfer intermediate with the first electron transfer as a limiting stage, accompanied by fast e^−^, H^+^ or e^−^, H^.^ transfer. This mechanism has been previously proposed for the reduction of NACs or quinones by NQO1 and other related two-electron (hydride) transferring flavoenzymes, whose reactivity tends to increase with an increase in the E^1^_7_ values of the compounds [23,36]. As compared to NACs, the higher reactivity of BFXs might be associated with their higher softness as a factor reinforcing their two-electron (hydride) reduction. In accordance with the maximal hardness principle ([29], and references therein), the transient reaction state, owing to its unstable system, must be much softer than the initial state of reactants. Thus one may suppose that for NQO1-catalyzed conversion of BFXs, less energy may be required for the formation of a charge transfer intermediate than for the reductive conversion of NACs. It has been previously defined that the reduction of NACs by NQO1 is characterized by more positive entropies of activation (∆S^#^) than those of highly reactive quinones ([23,37], and references therein). It can be linked to a more effective electronic coupling of quinones with the isoalloxazine ring of reduced FAD, as compared to that of NACs. In this study, we examined the temperature dependence of NQO1-catalyzed reduction of several more reactive BFXs. The data of quinone reductase, BFX-reductase, and nitro-reductase reactions of NQO1 are listed in Table 5, which shows that the reduction of BFXs is characterized by less negative entropies of activation than the reduction of quinones and by more negative entropies than the reduction of nitro-aromatic compound. The data suggest that during the reduction of BFXs by NQO1, these compounds may possess a more effective electronic coupling with the reduced FAD than that of NACs.

**Table 4:** Inhibition constants (K_i_) and the maximal degrees of inhibition (ε_max_) of NQO1 by BFXs and NACs. K_i_ values of NQO1 by NACs are taken from [20].

No.	Compound	K_i_ (µM)	ε_max_ (%)
	*Benzofuroxans*		
1.	Benzofuroxan	20.3 ± 3.9	72.9 ± 3.0
2.	5-Methylbenzofuroxan	25.7 ± 4.2	73.3 ± 2.5
3.	5-Dimethylbenzofuroxan	29.3 ± 2.5	87.0 ± 2.6
4.	5-Chlorobenzofuroxan	33.8 ± 4.2	82.0 ± 2.3
5.	5,6-Dichlorobenzofuroxan	22.1 ± 5.7	84.7 ± 4.2
6.	5-Methoxybenzofuroxan	32.9 ± 3.4	89.4 ± 1.2
7.	5,6-Dimethoxybenzofuroxan	55.3 ± 3.4	89.4 ± 1.2
8.	5-Trifluorobenzofuroxan	49.0 ± 2.8	77.0 ± 1.6
9.	5,6-Methylendioxybenzofuroxan	110 ± 19	75.9 ± 4.0
10.	5,6-Ethylendioxybenzofuroxan	112 ± 12	81.3 ± 2.8
11.	Benzodifuroxan	41.5 ± 5.0	100
12.	Benzotrifuroxan	46.0 ± 5.6	100
	*Nitrobenzenes*		
13.	Nitrobenzene	900	100
14.	1,2-Dinitrobenzene		
15.	1,3-Dinitrobenzene		
16.	1,4-Dinitrobenzene	200	100
17.	2,4,6-Trinitrotoluene	80	100

**Table 5:** The k_cat_ and k_cat_/K_m_ values of NQO1-catalyzed reduction benzo- and naphthoquinones, BFXs and 1,4-dinitrbenzene, and their activation enthalpies (∆H^≠^) and entropies (∆S^≠^).

Compound	k_cat_ (s^−1^)	k_cat_/K_m_ (M^−1^·s^−1^)	∆H^≠^ (kJ·mol^−1^)	∆S^≠^ (eu)
Tetramethyl-1,4-benzoquinone ^a^	1000 ± 90	6.7 ± 0.8 × 10^7^	4.44 ± 1.05	−76.09 ± 3.44
2-Hydroxy-1,4-nathphoquinone ^a^	232 ± 25	5.9 ± 0.5 × 10^6^	10.10 ± 3.35	−84.43 ± 8.30
Benzofuroxan	11.0 ± 1.5	8.4 ± 0.3 × 10^4^	30.45 ± 6.46	−42.52 ± 8.30
5,6-Dichlorobenzofuroxan	6.9 ± 0.1	1.3 ± 0.1 × 10^5^	30.87 ± 1.70	−46.06 ± 5.72
1,4-Dinitrobenzene ^b^	0.6 ± 0.1	2.3 ± 0.1 × 10^3^	50.58 ± 10.31	−8.81 ± 2.25

^a^ taken from [37]; ^b^ taken from [23].

#### 2.2.3. *o*-Benzoquinone Dioxime and 2,3-Diaminophenazine as the P-450R- and NQO1-Catalyzed Prime Reductive Intermediate and the Final Product of Benzofuroxan, Respectively

**Figure 8:**
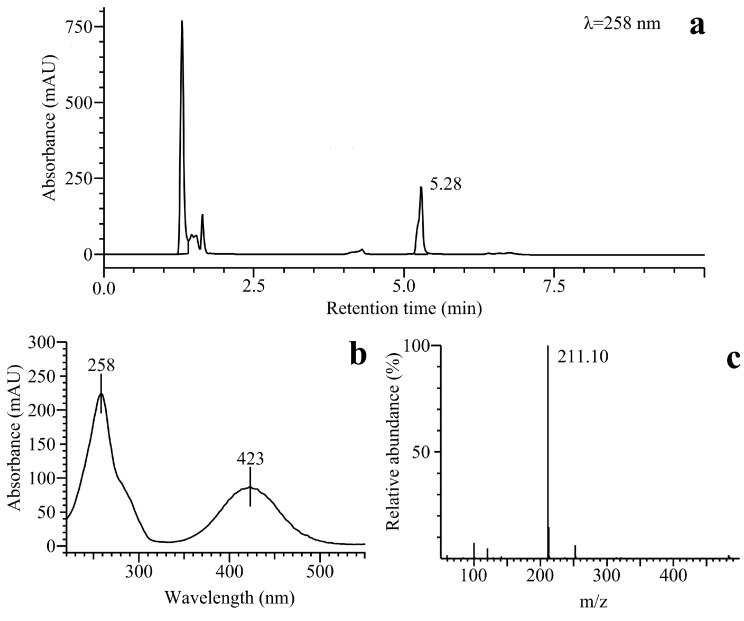
The liquid chromatography and mass spectrometry (LC-MS) analysis of the NQO1-catalyzed reduction product of benzofuroxan: (**a**) UV chromatogram of the reaction mixture; (**b**) UV-VIS absorbance spectrum of 2,3-diaminophenazine (PHADA); (**c**) MS-spectra (*m*/*z* of 211.10 consistent with [M+H]^+^ of PHADA).

**Figure 9:**
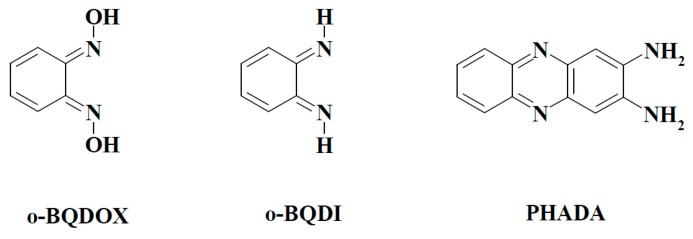
Structural formulas of the proposed reductive intermediates and the final product of benzofuroxan: *o*-benzoquinone dioxime (*o*-BQDOX), *o*-benzoquinone diimine (*o*-BQDI), and 2,3-diaminophenazine (PHADA)

We also performed a preliminary examination of the enzymatic reductive product(s) of benzofuroxan by means of liquid chromatography and mass spectrometry (LC-MS). The UV chromatogram of the NQO1-catalyzed reductive product of the compound revealed a distinct peak with a retention time of 5.28 min, as observed at 258 nm (Figure 8a). The UV-VIS absorbance spectrum exhibited two absorbance maxima at 258 and 423 nm (Figure 8b) and MS displayed m/z of 211 (Figure 8c), which is consistent with [MI+H^+^] of 2,3-diaminophenazine (PHADA) (the structural formulas of PHADA together with the predicted reductive intermediates of benzofuroxan are shown in Figure 9). PHADA was ascertained by its comparison with the high performance liquid chromatography (HPLC) retention time of its synthetic sample used as a standard, and ultimately confirmed by means of LC-MS (data not shown). Almost the same retention time, UV-VIS absorbance and mass spectra were obtained for the P-450R-catalyzed reduction of benzofuroxan (data not shown). As far as we know, this is the first direct confirmation of the formation of PHADA from the single- and two-electron transferring flavoenzyme-catalyzed reduction of benzofuroxan. One may note that PHADA has been previously identified as a metabolite of benzofuroxan in a cytosolic or microsomal fraction of rat liver [21], and as a product of its electrochemical reduction in aqueous media ([38], and references therein).

The reduction of benzofuroxan was proposed to proceed with the formation of *o*-benzoquinone dioxime (*o*-BQDOX) (Figure 9) as a primary two-electron reduced intermediate, which can be further reduced to o-benzoquinone diimine (*o*-BQDI) (Figure 9) by the four-electron transfer process yielding PHADA. It has been postulated that PHADA might be formed from the coupling of *o*-BQDI with o-BQDOX followed by a two-electron reduction and/or from the coupling of o-BQDI with itself ([38] and references therein).

Presuming that the enzymatic reduction of benzofuroxan may proceed through the formation of *o*-BQDOX as a primary two-electron reduced intermediate, we performed P-450R- and NQO1-catalyzed reductions of the synthetic sample of *o*-BQDOX. The UV-VIS spectrum of the reduction product, catalyzed by both flavoenzymes, was obtained to be analogous to that of the reduction of benzofuroxan, and the formation of PHADA was ultimately confirmed by LC-MS analysis (data not shown). We compared the enzymatic reactivity of benzofuroxan with that of *o*-BQDOX. The determined k_cat_/K_m_ values of reduction of *o*-BQDOX by P-450R and NQO1 were equal to 7.0 ± 0.6 × 10 ^4^ and 6.1 ± 0.5 × 10^5^ M^−1^·s^−1^, respectively, which were almost by an order of magnitude higher than the k_cat_/K_m_ values of the reduction of benzofuroxan (Table 2 and Table 3), showing that *o*-BQDOX is a more efficient oxidant of these flavoenzymes than benzofuroxan. This might be attributed to the preference of NQO1 and P-450R towards the oxidants of quinoidal structure ([15,37], and references therein). The redox-cycling ability of o-BQDOX was tested to be almost the same as that of benzofuroxan, *i.e.*, the reduction of *o*-BQDOX by both flavoenzymes was followed by slow O_2_ uptake, and the NQO1-catalyzed reduction of the compound was accompanied by the SOD-inhibited reduction of cytochrome *c* accounting for 30%–35% of NADPH oxidation rate. In the absence of NQO1, the direct reduction of cytochrome *c* by *o*-BQDOX did not occur.

Although in this study no direct evidence was obtained for the formation of *o*-BQDOX, PHADA identified from the reduction of benzofuroxan as well as from *o*-BQDOX enables us to suggest the possibility of the two-electron reduction of benzofuroxan to *o*-BQDOX as a prime intermediate, which can act as a much more efficient substrate undergoing a further reduction process yielding PHADA. The study of P-450R- and NQO1-catalyzed reductive intermediates and/or final products of other BFXs used in this study are beyond the scope of this work. It will be the subject of our forthcoming study. The study of the reactivity of BFXs, as well as related and/or hybrid structure compounds bearing BFX, towards these and other related flavoenzymes will be expanded in our future works.

## 3. Experimental Section

### 3.1. The Computational Details

The quantum mechanical calculations of BFXs and NACs were performed by PC Spartan ‘10 software package (Wavefunction Inc., version 1.1.0, 2011, Irvine, CA, USA). Their molecular structures were initially optimized using the mechanical force field (MMFF94) method. To define the low-energy conformers of BFXs bearing flexible substituents (Compounds 6 and 7), their conformational analyses were performed by using the conformational distribution module. Further computations were carried out using semi-empirical AM1 or PM6 method that was followed by B3LYP functional method in conjunction with 6-311+G(d,p) basis sets, applying RB3LYP and UB3LYP for initial (neutral) and single-electron reduced (anion radical) states, respectively. All the structures were globally optimized with symmetry restriction, and the stationary points of the optimized structures were confirmed by frequency analysis. For all the optimized structures, no imaginary frequencies were defined. In the UB3LYP calculation for anion radical states, the spin contamination was lower than 2.0%. The vertical (VEA) and the adiabatic (AEA) electron affinities of BFXs and NACs were assessed in terms of Gibbs free energy difference (at 298.15 K) according to the following definitions:

VEA = G° (optimized neutral state) − G° (anion state at optimized geometry of neutral state)

AEA = G° (optimized neutral state) − G° (optimized anion state)

The regional electrophilic Fukui function (F^+^_k_) values were assessed using B3LYP/6-311+G(d,p) according to the direct single point computation method as described by Contreras *et al.* [39], involving LUMO FMO coefficients and an overlap integral matrix between the orbital components. First, the Fukui functions for the orbital components (α) of the atomic orbital (µ) (F^α^_µ_) were computed as follows: $F_{\mu }^{\alpha }=\sum_{\nu }c_{\mu \alpha }c_{\nu \alpha }S_{\mu \alpha }=c_{\mu \alpha }^{2}+c_{\mu \alpha }\sum_{\nu \neq \alpha }c_{\nu \alpha }S_{\mu \nu }$ , where C_µα_ and C_να_ are µ-th coefficient and ν-th expansion coefficient, respectively, and S_µν_ is an overlap integral between the µ and ν orbital components. Next, the Fukui functions, condensed upon k atom of the compound (F^+^_k_), were obtained by summing F^α^_µ_( $F_{k}^{+}=\sum_{\mu ∍k}F_{\mu }^{\alpha }$ ) and checked for the normalization condition ( $\sum_{k}F_{k}^{+}=1$ ). These calculations were performed by employing the PYTHON open-source programming language.

### 3.2. Chemicals and Enzymes

Benzofuroxan was obtained from TCI (Tokyo, Japan). Mono-substituted BFXs (Compounds 1, 2, 4, 6, and 8) (Figure 1)) were synthesized by the direct one-step oxidation of corresponding *o-*nitroanilines by hypochlorite in the medium of pH 8.0–9.5 according to the method described in [40]. Di-substituted BFXs (Compounds 3, 5, 7, 9, and 10 (Figure 1)) were synthesized from the corresponding 1,2-dinitrobenzene derivatives using modified two-step methods [41]: (1) 2-nitrophenylazides were obtained by the nucleophilic substitution reaction; and (2) intermediates, azido/nitro- compounds underwent cyclization during the boiling of their solutions in acetic acid or toluene. Benzodifuroxan and benzotrifuroxan (Compounds 11 and 12 (Figure 1)) were synthesized by the cyclization of 1,3-diazido-2,4,6-trinitrobenzene and 1,3,5-triazido-2,4,6-trinitrobenzene, respectively, in trichloroacetic acid according to the method described in [42,43]. The reductive metabolites of benzofuroxan, *i.e.*, *o*-benzoquinone dioxime (*o*-BQDOX) and 2,3-diaminophenazine (PHADA) (Figure 9) were synthesized according to the methods described in [22,44,45]. The structure and purity of the compounds were checked by means of melting points, TLC, H-NMR, and UV-VIS and IR spectroscopy.

NADPH (AppliChem, Omaha, NE, USA), bovine cytochrome *c*, dicumarol (Sigma, St. Louis, MO, USA), glucose-6-phosphate and 2-methyl-1,4-naphthoquinone (menadione) (Aldrich), glucose-6-phosphate dehydrogenase, superoxide dismutase and catalase (Calbiochem, San Diego, CA, USA) were of the highest purity available and used as obtained.

Rat liver NADPH:cytochrome P-450 reductase (P-450R, EC 1.6.2.4) was prepared in accordance with the method described in ([46], and references therein) and its concentration was determined spectrophotometrically at 456 nm by using ∆ε_456_ = 21.4 mM^−1^·cm^−1^. NAD(P)H:quinone oxidoreductase (DT-diaphorase, NQO1, EC 1.6.99.2) was purified from rat liver according to the method as described by Prochaska [47], and its concentration was determined spectrophotometrically at 460 nm (∆ε_460_ = 11.0 mM^−1^·cm^−1^).

### 3.3. Enzymatic Assay and Estimation of Kinetic Data

The steady-state kinetic measurements of the enzymatic reduction of BFXs were carried out spectrophotometrically applying Lambda 25 (PerkinElmer, Waltham, MA, USA) or Carry 60 (Agilent Technologies, Santa Clara, CA, USA) UV-VIS spectrophotometers in 0.1 M K-phosphate buffer solution (pH 7.0) containing 1 mM EDTA, at 25 °C. The initial enzymatic rates were monitored according to NADPH oxidation at 340 nm (∆ε_340_ = 6.22 mM^−1^·cm^−1^), using 1.0 or 0.2 cm optical path cells, and corrected for the formation of 340 nm-absorbing products after recording their spectra in NADPH-regeneration system (10 U/mL glucose-6-phosphate dehydrogenase, 10 mM glucose-6-phosphate, and 15–20 μM NADPH). For certain BFXs, the reduction of the compounds was measured following NADPH oxidation at wavelengths of isosbestic points of their absorbance spectra, obtained in the NADPH-regeneration system, *i.e.*, at 305 nm (∆ε_305_ = 2.45 mM^−1^·cm^−1^) for 5-Cl-benzofuroxan, 350 nm (∆ε_350_ = 5.64 mM^−1^·cm^−1^) for 5-methoxy-benzofuroxan, 370 nm (∆ε_370_ = 2.60 mM^−1^·cm^−1^) for 5-ethoxybenzofuroxan, and 340 nm (∆ε_340_ = 6.22 mM^−1^·cm^−1^) for benzodifuroxan. The rates were additionally corrected for the intrinsic NADPH-oxidase activity of P-450R (0.11 s^−1^) and NQO1 (0.05 s^−1^). In separate experiments, the reduction of cytochrome *c* (50 μM) in the presence of NADPH and BFXs was measured spectrophotometrically according to an increase in absorbance at 550 nm by using ∆ε_550_ = 20.0 mM^−1^·cm^−1^. BFX-mediated oxygen uptake was monitored polarographically by Clark electrode (Digital Model 10, Rank Brothers Ltd., Cambridge, UK), at 25 °C.

The steady-state kinetic parameters, *i.e.*, the catalytic (k_cat_) and the apparent second-order rate (k_cat_/K_m_) constants of the enzymatic reduction of the compounds were determined fitting the data by nonlinear regression to the standard Michaelis-Menten expression (Equation (6)) and to its re-parameterized form (Equation (7)), for the calculation of k_cat_/K_m_ ratio as a separate parameter [48], where [E_0_] is the total enzyme concentration, *v* is the initial reaction rate, K_m_ is the apparent Michaelis constant, and [S] is the initial concentration of BFXs.

(6) $$\frac{v}{\left[E_{0}\right]}=\frac{k_{cat}[S]}{K_{m}+[S]}$$

(7) $$\frac{v}{\left[E_{0}\right]}=\frac{k_{cat}}{K_{m}}\left(\frac{\left[S\right]}{1+\frac{\left[S\right]}{K_{m}}}\right)$$

In separate situations, when [S] << K_m_, the k_cat_/K_m_ values were defined by linear regression (Equation (8)):

(8) $$\frac{v}{\left[E_{0}\right]}=\frac{k_{cat}}{K_{m}}[S]$$

The inhibition of NQO1 by BFXs was examined by measuring the initial rates of 2-methyl-1,4-naphthoquinone-mediated reduction of 50 μM cytochrome *c* at 550 nm (∆ε_550_ = 19.2 mM^−1^·cm^−1^), in the presence of 4 μM 2-methyl-1,4-naphthoquinone, 10–100 μM NADPH, and various fixed concentrations of BFXs as inhibitors. The action of BFXs as competitive inhibitors ([I]) with respect to NADPH oxidation, consistent with the enzyme operating under apparent second order condition in the reductive-half reaction of NQO1 [36], is represented by the minimal reaction scheme:

**Figure:**
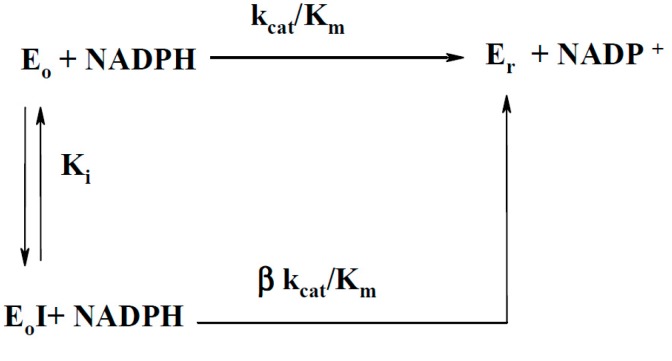


where E_o_ and E_r_ are the oxidized and reduced enzyme forms, respectively, k_cat_/K_m_ is the apparent second-order rate constant of NADPH oxidation, K_i_ is the inhibition constant, and β parameter represents the completeness of enzyme inhibition (0 ≤ β ≤ 1). Based upon this scheme, the hyperbolic dependence of k_cat_/K_m_, expressed as (k_cat_/K_m_)_0_/(k_cat_/K_m_) ratio on inhibitor concentrations is obtained (Equation (9)):

(9) $$\frac{{\left(k_{cat}/K_{m}\right)}_{0}}{\left(k_{cat}/K_{m}\right)}=\frac{K_{i}+[I]}{K_{i}+\beta [I]}=1+\frac{\varepsilon_{\max}[I]}{K_{i}+\beta [I]}$$

where k_cat_/K_m0_ is the second-order rate constant of NADPH oxidation in non-inhibited reaction ([I] = 0), and ε_max_(=1 − β) is the maximal degree of inhibition. In the case of the complete inhibition (β = 0 and ε_max_ = 1), Equation (9) is simplified to the standard linear expression (Equation (10)):

(10) $$\frac{{\left(k_{cat}/K_{m}\right)}_{0}}{\left(k_{cat}/K_{m}\right)}=1+\frac{\left[I\right]}{K_{i}}$$

In this study, the K_i_ and ε_max_ values were determined by fitting the experimental data to the standard hyperbolic expression of the inhibition degree (ε_I_) (=1 − (k_cat_/K_m_)/(k_cat_/K_m_)_0_) *versus* [I] (Equation (11)), which was obtained by the rearrangement of Equation (9), permitting a more accurate calculation of K_i_ and ε_max_, as compared to Equation (9).

(11) $$\varepsilon_{i}(\%)=(1-\frac{(k_{cat}/K_{m})}{{\left(k_{cat}/K_{m}\right)}_{0}})\times 100=\varepsilon_{\max}(\%)\frac{\left[I\right]}{K_{i}+[I]}$$

The enthalpies (∆*H*^≠^) and entropies *(*∆*S^≠^)* of the activation of the reduction of BFXs by NQO1 were defined from Eyring plots of ln [(k_cat_/K_m_)/T] *versus* 1/T, applying the k_cat_/K_m_ data obtained at seven fixed temperatures between 15 and 45 °C.

The estimation of the kinetic data and *QSAR* analysis were performed by using SigmaPlot 2000 (SPSS. Inc., version 6.10, Chicago, IL, USA) or Statistica (StatSoft Inc., version 8.0, Tulsa, OK, USA) packages.

### 3.4. Liquid Chromatography and Mass Spectrometry (LC-MS)

The reaction mixture of benzofuroxan reduction product(s) was extracted with ethyl acetate from the upper solution layer, dried by Na_2_SO_4_ and removed *in vacuo*. The residue was re-dissolved in acetonitrile and analysed by LC-MS system (CBM-20A controller, two LC-2020AD pumps, SIL-30AC auto sampler and CTO-20AC column oven; Shimadzu, Tokyo, Japan), equipped with a photodiode array (PDA) detector (SPD-M20A Prominence diode array detector, Shimadzu, Japan) and a mass spectrometer (LCMS-2020, Shimadzu, Japan), equipped with electrospray ionization (ESI) source. The chromatographic separation was conducted by using YMC-Pack Pro C18 column, 4 mm × 150 mm (YMC, Kyoto, Japan) at 40 °C and mobile phase, consisting of 5 mM ammonium acetate, pH 5.3 (Solvent A) and acetonitrile (Solvent B), delivered in a gradient elution mode at a flow rate of 0.6 mL·min^−1^. The following elution program was used: 0–0.5 min, 5% B; 0.5–5 min, 60% B; 5–5.1 min, 60% B; 5.1–5.2 min, 5% B; 5.2–10 min, 5% B. Mass scans were measured from *m*/*z* 10 to *m*/*z* 700, at 350 °C interface temperature, 250 °C DL temperature, ±4.500 V interface voltage, neutral DL/Qarray, using N_2_ as nebulizing and drying gas. Mass spectrometry data were acquired in both positive and negative ionization modes. The data were analyzed by LabSolutions LC-MS software.

## 4. Conclusions

The results of this work show that BFXs act as relatively efficient substrates for single-electron transferring flavoenzyme P-450R as well as for two-electron transferring flavoenzyme NQO1, which are important targets for various redox-active xenobiotics and drug agents. The reduction of BFXs by both enzymes proceeded with the concomitant formation of their reduction product(s) accompanied by marginal O_2_ uptake, except for annelated BFXs, whose reductive intermediates were rapidly re-oxidized by O_2_ producing peroxide. The reactivity of BFXs towards single-electron transferring P-450R increased upon an increase in their electron-accepting potency, showing almost the same dependence as NACs. This may suggest that the tentative self-exchange rate constant (k_ex_) of BFXs might be close to that of NACs, k_ex_ = 10^4^–10^5^ M^−1^·s^−1^ [49]. In NQO1-catalyzed two-electron (hydride) transferring reactions, BFXs acted as more efficient substrates than NACs, and the reductive conversion of BFXs by two-electron (hydride)-transferring NQO1 was markedly higher than by single-electron transferring P-450R. The QSARs obtained in NQO1-catalyzed reactions imply that the reactivity of BFXs, as well as of NACs, is determined by their electron-accepting potency influenced by their binding mode in the active center of NQO1 and affected by the electronic property of the compounds expressed in terms of their global softness index. It was found that the reductive conversion of benzofuroxan by both flavoenzymes yielded the same reduction product of benzofuroxan, 2,3-diaminophenazine, with the formation of *o*-benzoquinone dioxime as a putative primary reductive intermediate, which undergoes a further reduction process. The data of this study showed that in most cases the enzymatic reduction of BFXs does not initiate their redox-cycling, which may argue for a minor role of the redox-cycling-type action in the cytotoxicity of BFXs.
